# Ensemble Effects in Adsorbate–Adsorbate Interactions in Microkinetic Modeling

**DOI:** 10.1021/acs.jctc.2c01005

**Published:** 2023-01-18

**Authors:** Elisabeth M. Dietze, Henrik Grönbeck

**Affiliations:** Department of Physics and Competence Centre for Catalysis, Chalmers University of Technology, SE-412 96Göteborg, Sweden

## Abstract

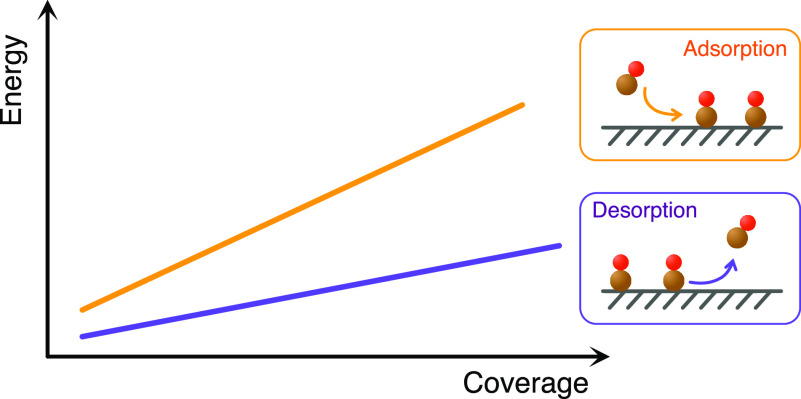

Adsorbates on a surface experience lateral interactions that result in a distribution of adsorption energies. The adsorbate–adsorbate interactions are known to affect the kinetics of surface reactions, which motivates efforts to develop models that accurately account for the interactions. Here, we use density functional theory (DFT) calculations combined with Monte Carlo simulations to investigate how the distribution of adsorbates affects adsorption and desorption of CO from Pt(111). We find that the mean of the average adsorption energy determines the adsorption process, whereas the desorption process can be described by the low energy part of the adsorbate stability distribution. The simulated results are in very good agreement with calorimetry and temperature-programmed desorption experiments and provide a guideline of how to include adsorbate–adsorbate interactions in DFT-based mean-field kinetic models.

## Introduction

Reactions over solid surfaces are commonly modeled by solving the chemical master equation using either mean-field or kinetic Monte Carlo approaches.^1−3^ The reaction kinetics in the mean-field approach are determined by coverages, and the underlying assumption is a uniform surface with ideal adsorbate mixing. Surface reactions in applications, such as heterogeneous catalysis, are generally performed at elevated temperatures, which implies rapid adsorbate diffusion and a high degree of adsorbate mixing. The kinetic Monte Carlo method is instead based on propagation of surface states with an explicit adsorbate distribution.

**Figure 1:**
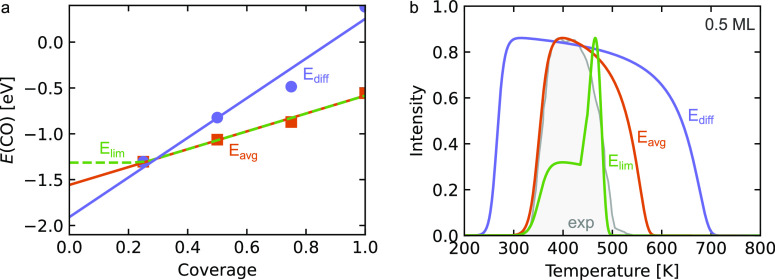
(a) Mean adsorption energy (orange) and differential adsorption energy (violet) vs coverage obtained from a 2 × 2 supercell. The lines are linear fits. The green line shows a constant mean adsorption energy for θ ≤ 0.25. (b) TPD obtained using the Redhead formula for θ = 0.5 ML and the fit from the points in panel a (β = 15.5 K, ν = 2 · 10^14^ s^–1^). Experimental values (gray) taken from Steininger et al.^14^

The understanding of surface reactions has evolved during the past decades partly thanks to the possibility to perform kinetic modeling based on first-principles calculations.^4^ The standard procedure has become to use density functional theory (DFT) to calculate the relevant adsorption and reaction energies. The entropies of the surface species are generally obtained within the harmonic approximation to include temperature and pressure effects.^5,6^ However, one challenge in first-principles based kinetic modeling is the inclusion of adsorbate–adsorbate interactions. The adsorbate–adsorbate interactions affect the stability of the surface species and, thus, the reaction kinetics. To obtain the adsorbate–adsorbate interactions for mean-field kinetic modeling, typically different surface cells and coverages are used to describe the adsorption energy as a function of coverage. The adsorption energies are usually calculated as the average (*E*_avg_) or the differential adsorption energy (*E*_diff_):
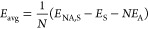
1
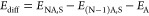
2*N* is the number of adsorbates on the surface, *E*_NA,S_ is the total energy of the surface with adsorbates, *E*_S_ is the total energy of the bare surface, and *E*_A_ is the total energy of the adsorbate in the gas phase. The *E*_avg_ and *E*_diff_ for CO adsorption on Pt(111) are shown in Figure 1a. Because of the uncertainty in the absolute DFT values, we have to apply a correction scheme suggested by Abild-Pedersen and Andersson.^7^ (Coverage dependencies should not be as dependent on the choice of exchange-correlation functional as the absolute adsorption energy.^8,9^)

The data points in Figure 1a are fitted using linear functions, which for the average adsorption energy is
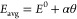
3α is the slope, and the adsorption energy at zero coverage is *E*^0^. It is clear that the slope of the differential adsorption energy is higher than that of the average adsorption energy. It should be noted that the adsorbate–adsorbate interactions can be fitted with other functional forms, such as exponential^6^ or polynomial^10,11^ functions. The differential adsorption energy can also be calculated from the internal energy (*E*_int_) according to^10^
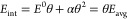
4
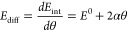
5This analysis shows that the differential adsorption energy should have a slope twice that of the average adsorption energy.

One question is whether *E*_avg_ or *E*_diff_ should be used in kinetic mean-field models. As *E*_diff_ is the energy of the least bound adsorbate on the surface, it seems reasonable that the *E*_diff_ value should be used as done in refs (12) and (13). One way to assess the adsorbate–adsorbate interactions used in models is to compare simulated results with surface science experiments performed under well-defined and clean conditions. Experimentally, coverage dependent desorption and adsorption energies can be studied employing temperature-programmed desorption (TPD) or single crystal adsorption calorimetry (SCAC) measurements. Focusing on TPD, the mean-field expression is given by the Redhead formula:
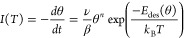
6ν is the prefactor, β is the heating rate, θ is the coverage, *n* is the order of the reaction, and *E*_des_(θ) = −*E*_ads_ = −*E*^0^ – γθ is the coverage dependent desorption energy. Note that the adsorption energy usually is defined as a negative number, whereas the desorption energy generally is defined as a positive number. The absolute adsorption and desorption energies are equal in the limit of low or full coverages and absence of activation barriers. Using the fitted coverage dependencies in Figure 1a and eq 6, we obtain the TPD spectra in Figure 1b. The prefactor contains information about entropy changes, and we have used a value close to the results of the hindered and free translator model for adsorbed CO,^6^ which shows good agreement with CO oxidation experiments. The simulated spectra are compared to the experimental TPD measurement by Steininger et al.^14^ The choice of how the adsorbate–adsorbate interactions are included has considerable effects on the simulated TPD spectra. Using the differential adsorption energy results in CO desorbing at 250 K, whereas the desorption starts at about 325 K using the average adsorption energy. Using a constant adsorption energy at low coverages results in a spectrum with multiple features. Comparing the simulated spectra to the experimental TPD measurement, we find that the onset of desorption is offset by 75 K to lower temperatures for the differential adsorption energy, whereas it is close for the average adsorption energy. Furthermore, the temperature range of desorption is severely overestimated using either the differential or average adsorption energy. The simulation with the constant low-coverage adsorption energy has a reasonable desorption width; however, the double feature is not present experimentally.

The poor agreement between the simulated and measured TPD spectrum for the generic case of CO adsorption on Pt(111) has motivated us to explore the adsorption–adsorption interactions in some detail. It is clear that important aspects are neglected using the standard approaches to include adsorbate–adsorbate interactions in mean-field models. In the present work, we develop a methodology to take the ensemble effects in the adsorbed CO layer into account. The methodology is based on a parametrized interaction model, which is used in Monte Carlo simulations to investigate the coverage dependent distribution of adsorption energies. The statistical data obtained from Monte Carlo simulations is used to obtain effective interaction parameters, which are used to simulate TPD spectra and adsorption energies as measured in SCAC measurements. The simulated TPD spectra and adsorption energies are in very good agreement with experimental data when the ensemble effects in the adsorbate layer are taken into account.

## Computational Methods

### First-Principles Calculations

We consider various adsorption structures for CO on Pt(111) to calculate adsorption energies. A detailed account of the structures are given in the Supporting Information (SI). All energies are obtained using density functional theory (DFT) as implemented in VASP 5.4.^15−17^ The calculations are performed using the PBE^18^ functional. The interactions between cores and valence electrons are described using the plane augmented wave method,^19,20^ and the Kohn–Sham orbitals are expanded using a 450 eV cutoff energy. The employed lattice constant for Pt is 3.967 Å. A Monkhorst–Pack scheme^21^ is used to sample the Brillouin zone. The *k*-point densities are given in Table S1. All surface slabs consist of four atomic layers, of which the two bottom layers are fixed to the bulk positions. The slabs are separated by at least a 10 Å vacuum in the *z*-direction. Structures are considered converged when the force per atom is below 0.01 eV/Å and the total energy difference between steps are lower than 10^–7^ eV. To correct for the differences in adsorption site preference between DFT calculations and experiments, we add a correction (Δ*E*_c_) to the adsorption energy based on the C–O stretch vibration (ω_CO_):^7,22^
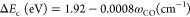
7The correction accounts for the overbinding of CO to metal surfaces using the PBE functional and, in particular, the too strong 2π*-backbonding in bridge and hollow sites.

### Interaction Model

The adsorption energy for a CO molecule at a given coverage is described as the sum of the low-coverage adsorption energy (θ = 1/9 ML) and repulsion terms. To parametrize the repulsive CO–CO interactions, we assume that repulsions on Pt(111) can be described as a sum of the distance-dependent direct CO–CO repulsion *V*_gas_ and a metal mediated repulsion *V*_metal_:
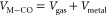
8This type of separation of interactions should be valid at coverages below ∼0.5 ML. At higher coverages, CO has been measured to occupy also hollow sites and to be displaced from the regular adsorption sites,^23^ which is not considered in our model. In addition, coverages higher than 0.5 ML on Pt(111) represent a surface poisoned state, which is less important for surface reactions.

**Figure 2:**
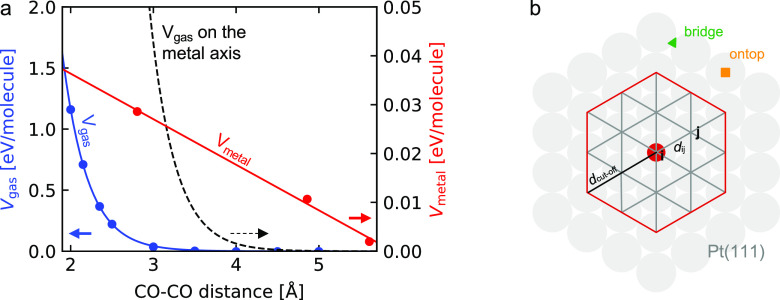
(a) *V*_gas_ (blue, left axis) and *V*_metal_ (red, right axis) in electronvolts per molecular neighbor depending on the CO–CO distance in Å. The black dashed line represents *V*_gas_ on the same axis scale as *V*_metal_. (b) Schematic of a Pt(111) surface with selected atom *i* and sample neighbor top site *j* connected by distance *d*_*ij*_. All sites within and on the red line are considered for the interactions of site *i*.

To calculate the repulsion terms for one CO molecule (CO^*i*^) at given coverage, we determine the distance to other CO molecules on the surface (CO^*j*^). The distance between the molecules defines the strength of *V*_gas_ and *V*_metal_, respectively. The total repulsion for CO^*i*^ can be described as
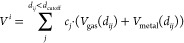
9The sum runs over surface sites within a cutoff distance, *d*_cutoff_ = 5.61 Å. *c*_*j*_ is 0 or 1, indicating whether the site *j* is occupied or not. *d*_*ij*_ is the distance between the adsorption sites *i* and *j. V*_gas_ is obtained from the distance-dependent repulsion between two parallel CO molecules in a large vacuum box as shown in Figure 2a. *V*_gas_ can be fitted with an exponential function (*a*_g_ · exp(−*b*_g_ · *d*_ij_)) with *a*_g_ = 866.45 eV and *b*_g_ = 3.31 1/Å. After determining *V*_gas_, *V*_metal_ is calculated as the difference between the adsorption energies as obtained in the DFT calculations and the model adsorption energy. The resulting linear distance dependence for *V*_metal_ is also shown in Figure 2a. The fitted values for *V*_metal_ = *a*_metal_*d*_ij_ + *b*_metal_ are *a*_metal_ = −0.009 eV/Å and *b*_metal_ = 0.055 eV. *V*_gas_ dominates for distances below 3.2 Å, whereas *V*_metal_ dominates for large distances. We do not observe interactions for distances larger than 5.6 Å, which defines the cutoff distance. Figure 2b visualizes the considered interaction area with *d*_*ij*_ and *d*_cutoff_.

### Monte Carlo Simulations

To evaluate the energy distribution for different adsorbate configurations of CO on Pt(111), we employ a Monte Carlo model. A 4 × 4 surface cell is used to sample the different surface configurations. Two possible site occupations are considered, namely, atop and bridge. The energy of the system is calculated using the interaction model. A new configuration is obtained by swapping the occupation of two randomly selected sites. The change in occupation is accepted when either the new energy *E*_new_ is lower than the current energy *E*_curr_ or the following condition is fulfilled:
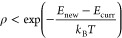
10ρ is a uniformly distributed random number between 0 and 1, *k*_B_ is the Boltzmann constant, and *T* is the temperature. To analyze the coverage dependent energy distribution, all atomic configurations with *E*_avg_ < 0 are used. The model is initialized with the configuration corresponding to the lowest average energy in the DFT calculations. Results using larger surface cells are shown in the Supporting Information.

## Results

**Figure 3:**
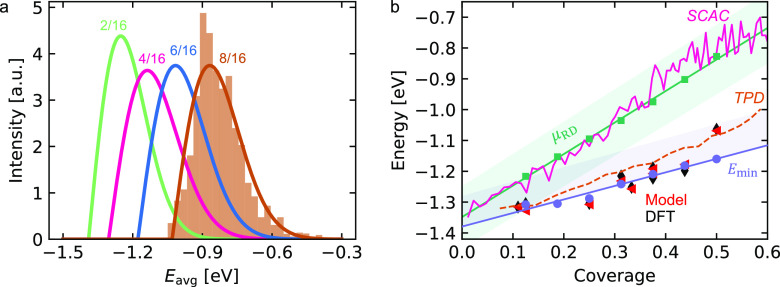
(a) Fitted Rayleigh distribution of *E*_avg_ for *N* = 2–8 CO molecules on a 4 × 4 supercell obtained with Monte Carlo simulations with *T* = 200 K. The histogram shows the raw data for *N* = 8 from the Monte Carlo simulation. (b) Coverage dependent adsorption energy. Experimental data from Fischer-Wolfarth et al.^24^ (SCAC) and Kelemen et al.^25^ (TPD) as solid and dashed lines in pink and orange, respectively. Results obtained with DFT calculations and the interaction model are shown with black diamonds and red triangles, respectively. Results from the Monte Carlo simulation: violet, *E*_min_ (circles) with fitting line, dark green, μ_RD_ (squares) with fitting line. Shaded area represents standard deviation obtained from the Monte Carlo simulation.

Having access to a parametrized interaction model makes it possible to study the energy distribution of adsorbates on a surface and the dependence of the energy distribution on coverage and temperature. Figure 3a shows the distribution of the average adsorption energy of CO on a 4 × 4 Pt(111) surface for a coverage of 0.5 ML. To ensure thermodynamic stability, a temperature of *T* = 200 K was chosen during the Monte Carlo simulation. The average adsorption energy is calculated as the total adsorption energy of a configuration divided by the number of adsorbates. The distribution is asymmetric and has a width of about 0.5 eV. The highest average adsorption energy is about −0.5 eV. To conveniently describe the obtained energy distribution, we fitted the Monte Carlo data with a Rayleigh distribution (solid line). The Rayleigh distribution describes the distance between the origin and two spacial coordinates (*x*, *y*) given that the coordinates are normally distributed with an average value of zero. We have chosen the Rayleigh distribution, as the leading interaction at large distances has a linear distance dependence (*V*_metal_). (For comparison, we also fitted the data to a Maxwell distribution. The results are similar, and the corresponding plots are shown in Figures S2 and S3 in the SI.)

Figure 3a includes also the fitted distributions for three lower coverages. A lowering of the coverage results in a shift to lower energies, whereas the width does not show any obvious coverage dependence. The distribution with the result from the Monte Carlo simulation is reported in Figure S2. The Rayleigh distribution is described by two parameters: *s*, the scaling, and *l*, the location. *l* is the shift of the distribution relative to zero. The mean μ_RD_ of the energy and the standard deviation σ_RD_ are given by
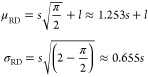
11

The coverage dependent mean energies obtained from the Rayleigh distributions are shown in Figure 3b. The standard deviation is similar for all coverages and is shown as a shaded area. Figure 3b shows also the coverage dependence of the minimum energy *E*_min_ (strongest CO–surface bond) including the standard deviation toward higher energies. The minimum energy is calculated using the most stable structure for each coverage. For comparison, the results obtained from the DFT calculations used to parametrize the interaction model together with the corresponding results by the interaction model are shown as black diamonds and red triangles, respectively. The agreement between the DFT calculations and the interaction model is very good as shown also by a parity plot (Figure S1 in SI). The calculated DFT values show no clear trend for coverages below θ = 0.2 ML. This is in agreement with a previous report by Grabow et al.^12^ showing a constant average adsorption energy at low coverages. In our interaction model, we use a constant adsorption energy for θ < 0.11 ML. The coverage dependencies obtained from the Monte Carlo simulations show linear coverage dependencies for μ_RD_ and *E*_min_ for θ ≥ 2/16 ML. However, the slopes are clearly different, with the largest slope for μ_RD_. The slopes and intersections with the energy axis are summarized in Table 1.

**Table 1:** Coverage Dependent CO Adsorption Energy on Pt(111) Assuming a Linear Energy Dependence^a^

origin	method	coverage	*E*^0^	slope
μ_RD_ (200 K)	MC	0.13–0.50	–1.34	1.02
*E*_min_ (200 K)	MC	0.13–0.50	–1.38	0.44
Fischer-Wolfarth et al.^24^	SCAC	0–0.64	–1.29	1.02
Yeo et al.^26^	SCAC	0–0.58	–1.94	1.31
Hörtz et al.^27,28^	SCAC	0–0.5	–1.37	1.76
Campbell et al.^29^	TPD	0–0.4	–1.42	0.37
Kelemen et al.^25^	TPD	0–0.44	–1.33	0.61
Steininger et al.^14^	TPD	0.17–0.58	–1.50	0.55

a Given are the origin, the employed method, fitted coverage range in ML, *y* intercept (*E*^0^ [eV]), and slope in eV/ML.

To compare our calculated coverage dependencies to experiments, we are considering both TPD spectra and heat of adsorption measurements. During TPD, the desorption probability of CO from the surface is measured. The surface is initially covered with a certain amount of CO. A constant heating rate is applied, and the number of CO molecules desorbing is measured. The heating rate is generally low enough for the system to anneal to low energy states for the considered coverage. However, due to the dynamic behavior of the CO molecules on the surface, also configurations with higher energy could contribute to the measurement. During the heat of adsorption measurements, the heat released upon adsorption of CO on the Pt(111) surface is measured. The initial surface is empty, and due to continuous pulses of CO, the surface coverage is increased. The position on the surface where the CO molecule impinges can be assumed to be random. The CO molecule either impinges at a location with low or high local coverage. Depending on the local coverage, the heat of adsorption could vary. Therefore, a large part of the distribution is probed. The zero-coverage limit of the adsorption and desorption energy should coincide as the processes are nonactivated for the considered system. For example, the *E*^0^ obtained by Fischer-Wolfarth et al.^24^ in calorimetry measurement is close to the value reported from TPD measurements by Kelemen et al.^25^ (Table 1). It should be noted that the desorption energy obtained by TPD experiments depends on the choice of prefactor in the analysis.

Even if the *E*^0^ values coincide for the two types of experiments, it is clear that the coverage dependencies (slopes) are different. The slopes obtained in the TPD measurements are less steep than the slopes measured in calorimetry. Comparing the experiments with the simulations, we find a good agreement between the calorimetry measurements by Fischer-Wolfarth et al.^24^ and the slope of μ_RD_, which is 1.0 eV/ML. The slopes obtained by Yeo et al.^26^ and Hörtz et al.^27,28^ are steeper. The difference in slopes for the calorimetry measurements could be related to pulse rate and intensity as well as different temperatures, although all data are reported to be at 300 K. Hörtz et al.^28^ showed that the slope is sensitive to the temperature.

**Figure 4:**
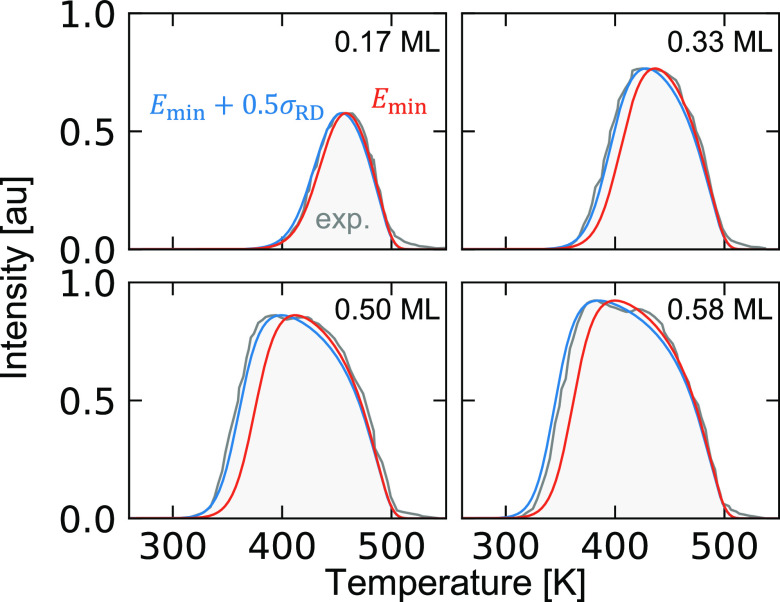
TPD spectra for the coverages: 0.17, 0.33, 0.5, and 0.58 ML measured by Steininger et al.^14^ The red curve uses the slope obtained from the minimum energy, and the blue curve includes 0.5σ_RD_ at 0.5 ML. The heating rate is β = 15.5 K and the prefactor ν = 2 × 10^14^ s^–1^.

We also find a good agreement with the TPD measurements^14,25,29^ when the slopes are obtained from *E*_min_, which is 0.44 eV/ML. A direct comparison to the TPD data from Steininger et al.^14^ is shown in Figure 4 for four coverages. We find that the slope obtained using *E*_min_ is slightly underestimating the adsorbate interactions, in particular at the higher coverages. Owing to the dynamic character of the adsorbed-layer, it is unlikely that the system is in the configuration with minimum energy, instead an ensemble of structures is probed. Therefore, we include also the result obtained with 0.5σ_RD_ at 0.5 ML. The resulting TPD spectra agree very well with the experimental spectrum.

We conclude that the mean adsorption energy (eq 11) obtained from the MC simulation can be used to describe the coverage dependence of the adsorption energy measured in calorimetric experiments. The energies measured in the TPD experiments are instead described by the tail of the energy distributions toward the minimum energy configuration.

## Conclusions

Procedures to construct mean-field kinetic models from DFT data have been established during the past decade. One challenge is still, however, to accurately describe adsorbate–adsorbate interactions. Different functional forms of the coverage dependence of adsorption energies have been used in the literature, based on either average or differential adsorption energies. To guide the construction of the models for adsorbate–adsorbate interactions, comparisons could be made to TPD and calorimetry measurements on well-characterized surfaces. Taking CO on Pt(111) as an example, we have in the present work outlined a scheme to parametrize adsorbate–adsorbate interactions based on DFT calculations. Adsorption energies from DFT calculations were used to construct an interaction model, which was applied in Monte Carlo simulations. The Monte Carlo simulations were used to investigate the statistical distribution of adsorption energies of CO molecules on Pt(111) as a function of coverage. We found that the distribution of adsorption energies could be described by a Rayleigh distribution. An analytical distribution gives access to the minimum and mean adsorption energy as well as the standard deviation. Comparing the simulated results with TPD and heat of adsorption measurements, we find that TPD spectra are very well represented by the minimum *average* adsorption energy including half the standard deviation. The heat of adsorption experiments are instead represented by the mean average adsorption energy. We note that the differential adsorption energy, which is sometimes used to model adsorbate–adsorbate interactions, does not describe either the TPD or the calorimetry measurements. The results for the slopes match the coverage dependence measured using TPD and calorimetry measurements and show that adsorbate–adsorbate interactions, in principle, should be modeled differently for adsorption and desorption/reaction events. A difference between adsorption and desorption is explicit in kinetic Monte Carlo simulations, whereas the coverages of the two states could be connected by a diffusion step in mean-field models. The outlined scheme to obtain accurate adsorbate–adsorbate interactions is general and can be extended also to cases with different types of adsorbates on the surface. In cases with repulsive interactions and known adsorption sites, a simple interaction model as presented here could be applicable. For adsorbates with attractive/mixed interactions, the interactions are best described using cluster expansion within the Monte Carlo simulations to obtain the effective adsorbate–adsorbate interactions.

## Supporting Information Available

Numerical results for the DFT calculations and interaction model, fitting parameters for coverage dependencies, distribution plots for different coverages, fitting parameters for the Rayleigh and Maxwell distributions, and comparisons of adsorbate stabilities using the interaction model with different surface cells (PDF)
An ASE database file for the considered structures (ZIP)
