# Supplementary material for: Ensemble Effects in Adsorbate–Adsorbate Interactions in Microkinetic Modeling
Source: J Chem Theory Comput. 2023 Jan 18;19(3):1044–9. doi: 10.1021/acs.jctc.2c01005 (PMC9933425; doi:10.1021/acs.jctc.2c01005)
Supplement: Supplementary file 1 — ct2c01005_si_001.pdf [file ct2c01005_si_001.pdf]

Supporting Information: Ensemble effects in adsorbate-adsorbate interactions in micro-kinetic modeling

Elisabeth M. Dietze* and Henrik Grönbeck*

Department of Physics and Competence Centre for Catalysis, Chalmers University of Technology, Göteborg, Sweden.

E-mail: dietze@chalmers.se; ghj@chalmers.se

DFT and model energies

The calculated DFT energies and the energies as obtained by the parameterized model are shown in Table S1. A parity plot is presented in Fig. S1 to facilitate the comparison. The energies in Table S1 and Fig. S1 are reported without any correction used in the main text to account for issues with the exchange-correlation functional. The first column in the table reports the considered surface cell and the position of the CO molecules (top, t-top, b-bridge, f-fcc hollow and h-hcp hollow). Due to the large configurational space in cases with large surface cells and multiple adsorption sites, only two and three different configurations are considered for each case (v1, v2, v3). The structures including CO adsorption in fcc and hcp positions are included to demonstrate that the parameterized interaction model reproduces the correct overall trend of the adsorption energies although fcc and hcp sites were not included in the parameterization.

Table S1: Surface cell, adsorption sites, coverage, total number of CO molecules on the surface (N), DFT energy for slab with adsorbates ($E_{\mathrm{NA,S}}$), energy of only the surface slab (E_{S}), average adsorption energy (E_{avg}) and the corresponding interaction model energy (E_{Model}). All energies are in eV. The CO gas-phase reference is -14.780 eV.

Structure	Coverage	N	$E_{\mathrm{NA,S}}$	E_{S}	E_{avg}	E_{Model}
(3×3) top	0.11	1	-224.693	-208.246	-1.667	-1.667
(2×2) top	0.25	1	-108.941	-92.505	-1.656	-1.659
(2×2) top	0.5	2	-124.895	-92.505	-1.415	-1.420
(2×2) top	0.75	3	-140.511	-92.505	-1.222	-1.181
(2×2) top	1	4	-155.257	-92.505	-0.908	-0.941
(2×4) top	0.125	1	-201.794	-185.359	-1.656	-1.665
(4×4) t3 b2	0.3125	5	-452.774	-370.719	-1.631	-1.647
(2×4) t1 b2	0.375	3	-234.641	-185.359	-1.648	-1.651
(2×4) t2 b1	0.375	3	-234.580	-185.359	-1.628	-1.612
(4×4) t4 b3	0.4375	7	-485.505	-370.719	-1.618	-1.598
(4×4) t3 b4 f1 h1 (v1)	0.5625	9	-517.500	-370.719	-1.529	-1.475
(4×4) t3 b4 f1 h1 (v2)	0.5625	9	-517.199	-370.719	-1.496	-1.407
(4×4) t3 b4 f1 h1 (v3)	0.5625	9	-517.568	-370.719	-1.537	-1.534
(4×4) t6 b2 h2 (v1)	0.625	10	-532.559	-370.719	-1.404	-1.287
(4×4) t7 b2 h1 (v2)	0.625	10	-532.415	-370.719	-1.390	-1.278

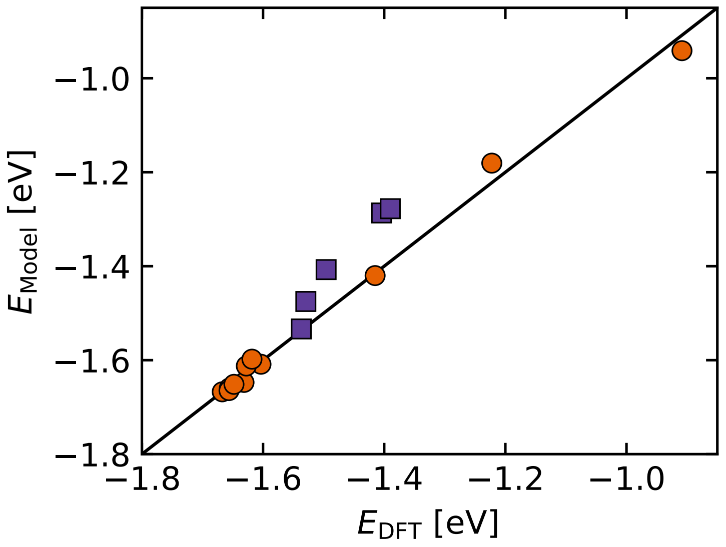

Figure S1: Parity plot of the calculated DFT values (E_{avg} in Table S1) and the corresponding values obtained with the interaction model (Table S1). Orange points: Structures which include only atop and bridge sites (MaxErr: 42 meV, MAE: 14 meV). Violet squares: Structures which include also hollow sites (MaxErr: 118 meV, MAE: 33 meV).

Table S2: Parameters used for the linear coverage dependence to obtain the TPD spectra in Figures 1 and 4 in the main text. The parameters are the slope γ (eV/ML) and y-axis intersection E^0 (eV). The E_{lim} parameters are used according to: ($\theta \leq 0.25 : E_{\text{lim}} = E^0 + \gamma \cdot 0.25$) and otherwise: $E_{\text{lim}} = E^0 + \gamma \cdot \theta$.

Figure	Fit	Slope γ	E^0
1	E_{diff}	2.16	-1.91
1	E_{avg}	0.97	-1.55
1	E_{lim}	0.97	-1.55
4	E_{min}	0.44	-1.38
4	$E_{\text{min}} + 0.5\sigma_{\text{RD}}$	0.56	-1.38

Table S3: Distance in Å and the corresponding interaction energy in eV/molecule for two parallel CO molecules in the gas-phase. (Data for Figure 2a in the main text.)

Distance [Å]	V_{gas} [eV/molecule]
2.0	1.160
2.15	0.710
2.35	0.367
2.5	0.222
3.0	0.036
3.5	0.002
4.0	0.000

Table S4: Distance in Å and corresponding energy in eV/molecule to fit V_{metal}. (Data for Figure 2a in the main text.)

Distance [Å]	V_{metal} [eV/molecule]
5.610	0.00197
4.859	0.01066
2.805	0.02859

Additional results from the Monte Carlo simulations

We are presenting the different distributions as obtained from the Monte Carlo simulations on a (4×4) surface cell at $T = 200$ K in Fig. S2 and at $T = 400$ K in Fig. S3. The histograms are fitted using Rayleigh and Maxwell distributions. The parameters of the fitted distributions are given in Tables S5 and S6. The obtained fits for the coverage dependencies of the mean value μ and standard deviation σ are summarized in Table S7.

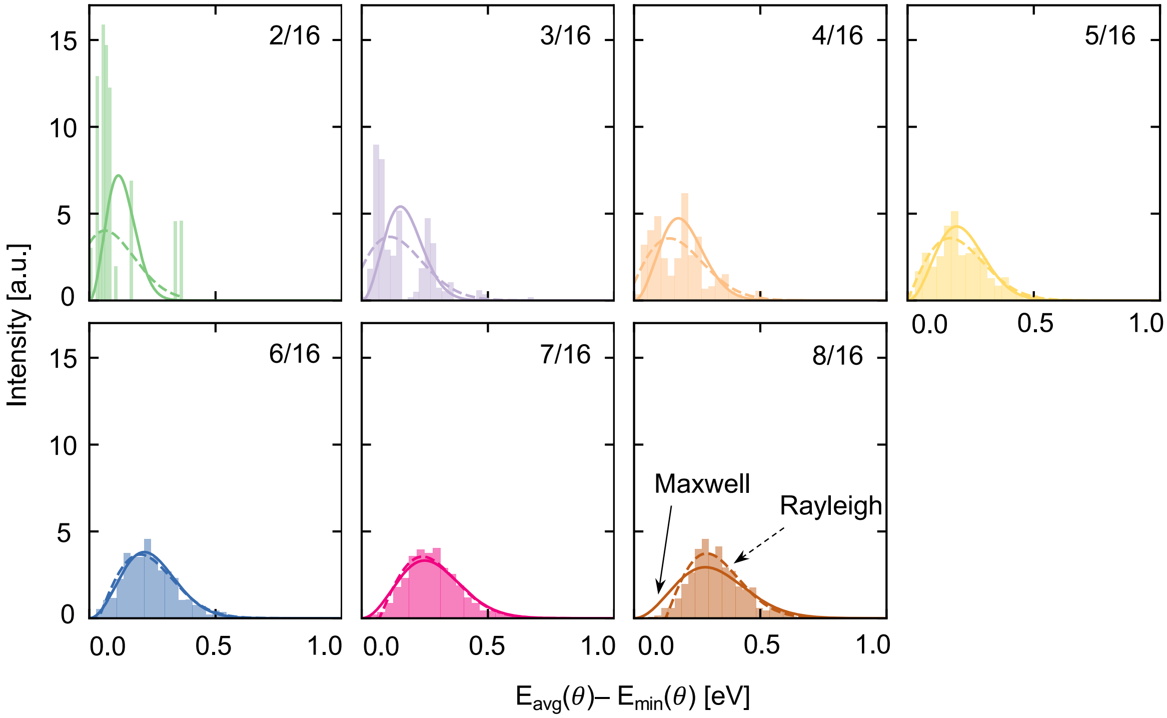

Figure S2: Distributions obtained from Monte Carlo simulations at $T = 200$ K with 30 bins each, fitted with either Maxwell (solid line) or Rayleigh (dashed line) distribution. The surface coverages are given in the left upper corner.

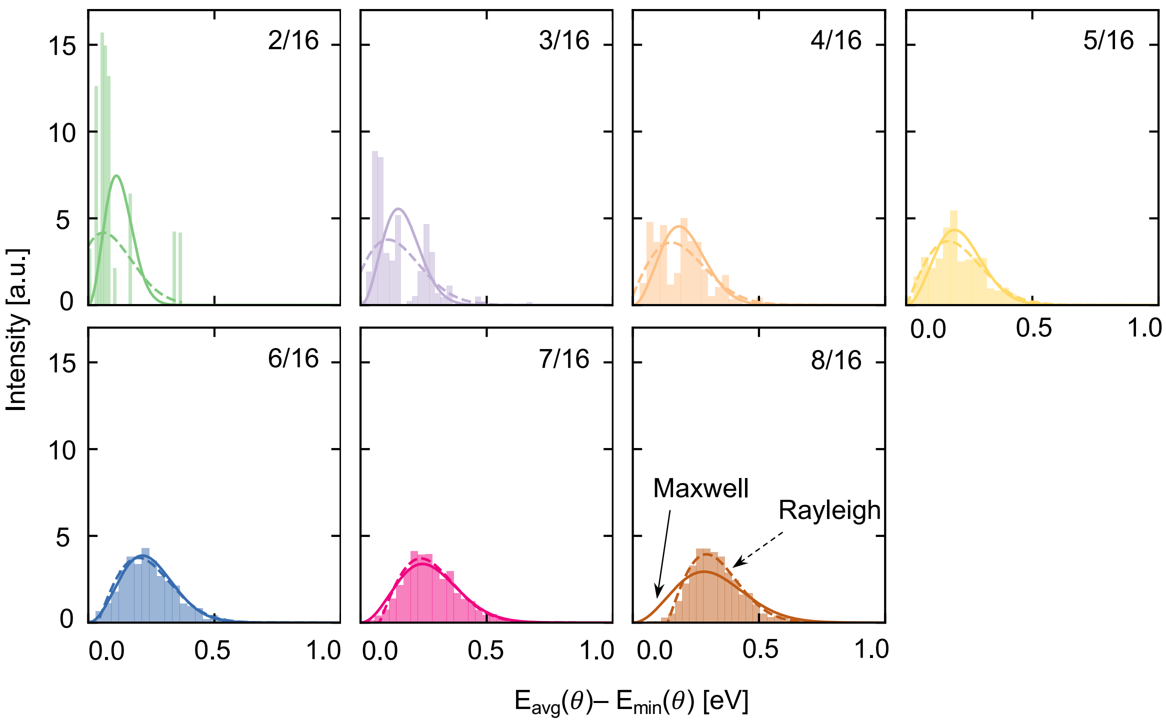

Figure S3: Distributions obtained from Monte Carlo simulations at T = 400 K with 30 bins each, fitted with either Maxwell (solid line) or Rayleigh (dashed line) distribution. The surface coverages are given in the left upper corner.

Table S5: Fitting parameters from the Monte Carlo data by applying a Rayleigh distribution. s is the scaling and l the location parameter as used also in Eq. (10) in the main text.

Coverage	$T = 200$ K	
	s	l
2/16	0.151	-1.390
3/16	0.165	-1.354
4/16	0.170	-1.303
5/16	0.169	-1.243
6/16	0.165	-1.178
7/16	0.171	-1.106
8/16	0.162	-1.030

Table S6: Fitting parameters from the Monte Carlo data by applying a Maxwell distribution. s is the scale parameter.

Coverage	$T = 200$ K
	s
2/16	0.081
3/16	0.108
4/16	0.124
5/16	0.138
6/16	0.154
7/16	0.176
8/16	0.200

Table S7: Fitting parameters obtained from a linear fit of the form $y = a \cdot x + b$ for the mean value (μ) of the Maxwell (MD) and Rayleigh (RD) distribution, respectively. The values are given in Tables S5 and S6.

Fitting	T	a	b	R^2
μ_{MD}	200	0.926	-1.311	0.9995
μ_{RD}	200	1.022	-1.338	0.9989

To investigate the effect of the applied surface cell size, we performed additional Monte Carlo simulations for (5×5), (6×6) and orthogonal (6×6) surface cells. The results are presented in Figure S4. The results show that μ_{RD} is well described using a (4×4) surface cell. E_{min} is for all cells similar in the case of $\theta \leq 0.4$ ML. For $0.4 \leq \theta \leq 0.5$, the (4×4) and orthogonal (6×6) cell give similar results. E_{min} obtained for the (5×5) and (6×6) cells are deviate from the linear fit, which is a consequence of that these cells not are commensurate with the stable c(4×2) overlayer structure.

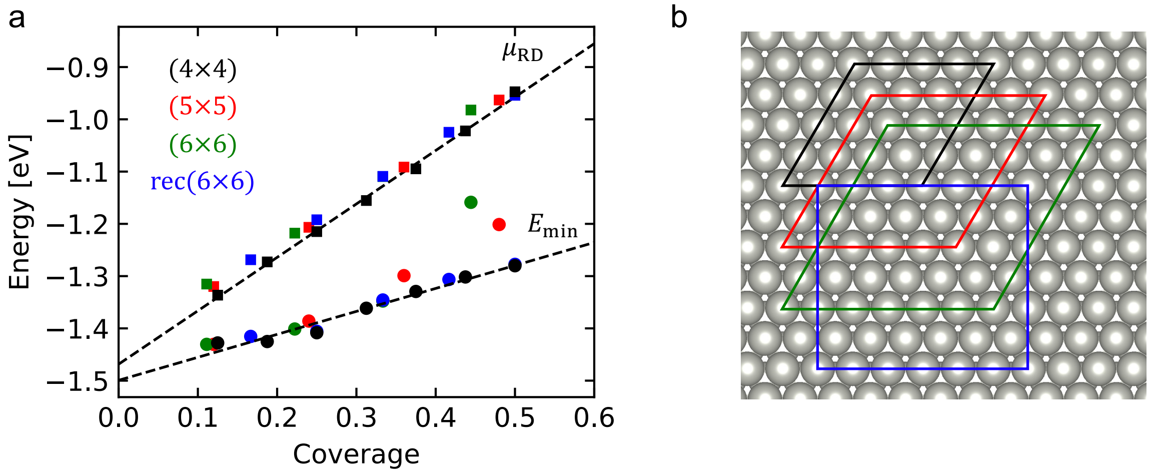

Figure S4: a: Coverage dependent energy obtained from Monte Carlo simulations of different cell sizes using the parameterized interaction model: black: (4×4), red: (5×5), green: (6×6) and blue: orthogonal (6×6). The black dashed lines give the fit for E_{min} (circles) and μ_{RD} (squares) of the black points. b: Applied surface-cells.
